# Effect of Concomitant Tricuspid Annuloplasty on Early Outcomes of Mitral Valve Replacement: A Study on Rheumatic Heart Disease Patients

**DOI:** 10.7759/cureus.13646

**Published:** 2021-03-01

**Authors:** Omer Farooq, Azam Jan, Usman Ghani, Amir Khan, Bahauddin Khan, Nabil I Awan, Hussain Shah

**Affiliations:** 1 Cardiothoracic Surgery, Rehman Medical Institute, Peshawar, PAK; 2 Surgery, Hayatabad Medical Complex, Peshawar, PAK

**Keywords:** mitral valve replacement, tricuspid annuloplasty, rheumatic heart disease

## Abstract

Introduction

Mitral valve abnormalities in rheumatic heart disease commonly lead to functional tricuspid regurgitation. Tricuspid annuloplasty (TA) is often performed in these cases along with mitral valve replacement (MVR). Our aim was to compare the perioperative morbidity and mortality among those patients that underwent mitral valve replacement with tricuspid annuloplasty versus those that underwent isolated mitral valve replacement.

Methods

A retrospective analysis of 158 patients that underwent mitral valve replacement, with or without tricuspid annuloplasty, secondary to rheumatic heart disease between January 2017 and August 2020. Patients who underwent additional cardiothoracic surgical procedures (aortic valve replacement and coronary artery bypass grafting) were excluded to reduce confounders.

Results

The study group consisted of 158 patients (mean age 41; 73 male, 85 female) that underwent MVR with TA (n=22; 13.9%) or without TA (n=136; 86.1%). Both groups had similar comorbidity frequencies and medication history. Preoperative echocardiography showed a comparable degree of pulmonary hypertension and ejection fraction between the two groups. The TA+MVR group had similar intraoperative (81.8% vs 66.9%; p=0.161) and postoperative (45.5% vs 45.6%; p=0.991) blood products usage compared to the MVR only group. Concurrent TA resulted in similar in-hospital mortality (4.5% vs 4.4%; p=0.977) as well as early postoperative complications, namely, prolonged ICU stay (13.6% vs 10.3%; p=0.639), prolonged ventilation (0 vs 2.2%; p=0.482), re-intubation (9.1% vs 2.9%; p= 0.161), and reopening for bleeding tamponade (0 vs 5.1%; p=0.276).

Conclusions

TA concurrently with MVR does not appear to increase in-hospital mortality or early postoperative complications.

## Introduction

Rheumatic fever and its sequelae continue to be abundantly prevalent in developing countries due to a lack of prompt identification and treatment of the causative agent - a group A streptococcal infection of the pharynx. The resulting autoimmune reaction targets multiple organs including the heart which is a frequent cause of significant morbidity and mortality. Although mitral valve involvement remains the most common aspect of endomyocardial involvement in rheumatic heart disease, concomitant tricuspid valve abnormalities are a frequent finding [1-2]. The pathogenesis behind tricuspid regurgitation (TR) observed in mitral valve disease remains complex. Often, the TR is functional secondary to the increased left atrial pressure caused by mitral valve disease [2]. Alternatively, the tricuspid dysfunction may be a product of independent involvement of the tricuspid valve by rheumatic heart disease [3]. Previously, it was postulated that functional TR may improve following mitral valve surgery as a result of improved hemodynamic parameters [4]. However, more recent studies have shown that TR may develop and progress despite correction of the left heart pathology and is likely associated with poorer outcomes in terms of morbidity and mortality [5-7].

Matsuyama et al. attempted to identify risk factors for the progression of TR following mitral valve surgery, identifying findings such as enlarged left atrium and atrial fibrillation as potential risk factors [8]. In view of the potential for TR progression, as well as late TR development, tricuspid annuloplasty (TA) may be considered for patients at the time of left heart surgery. This is especially true for patients with moderate-to-severe TR or significant dilation of the tricuspid annulus.

## Materials and methods

Patient selection and data acquisition

The target population of this study and their pertinent data were extracted from a maintained electronic database at the cardiothoracic surgery department of a tertiary care hospital. An established practice of initial paper-based collection of patient credentials and subsequent transfer to an Internet-based cloud system allows feasible data collection at our institute. The study was authorized by the research ethics committee. One-hundred fifty-eight patients that underwent mitral valve replacement (MVR) between January 2017 and August 2020 were included. Only patients who underwent concomitant tricuspid repair with MVR were retained in the study sample. One patient underwent tricuspid replacement with MVR, rather than tricuspid repair, and was excluded to preserve the homogeneity of the sample population. Exclusion criteria were set to filter out patients who had been subjected to additional cardiac procedures (such as aortic valve replacement and coronary artery bypass grafting) to limit any confounding variables and their effect on the final data analysis and results. The data were analyzed using the Social Package for the Social Sciences (SPSS; IBM Corp., Armonk, NY).

To calculate mean pulmonary artery pressure (mPAP) in these patients, the right ventricular systolic pressure (RVSP) was measured using preoperative echocardiography. RVSP = systolic pulmonary artery pressure (sPAP) in the absence of any right ventricular outflow tract obstruction. mPAP can then be calculated from the sPAP using the following equation [9]:

mPAP = 0.61 x sPAP + 2 mmHg

Prosthesis types

In the present age, the potential choices for mitral valve prosthesis are diverse, with seven mechanical prostheses and seven bioprostheses (6 porcine and 1 bovine) available to choose from [10]. As widely observed mechanical valves prove to be more durable, requiring fewer reinterventions at the expense of lifelong anticoagulation as well as higher bleeding and thrombosis risks.

In our sample of patients, 154 patients received mechanical valves and four patients received bioprosthetic valves.

Surgical techniques

All patients were operated on primarily for indications of mitral valve disease as per American Heart Association (AHA) guidelines. In all patients, mitral valve replacement was performed on-pump arrested heart with midline sternotomy. The approaches used to access the mitral valve varied between trans-septal and left atrial. Chordal preservation was the primary aim of each procedure. Tricuspid repair techniques in the sample population with concomitant tricuspid regurgitation varied between De Vega repair and Kay repair (bicuspidization) techniques.

The decision of tricuspid repair was made based on preoperative echocardiography and intraoperative evaluation, in accordance with the 2014 AHA/American College of Cardiology (ACC) guideline for the management of patients with valvular heart disease [11]. According to these guidelines, the indications for tricuspid valve surgery in patients undergoing left-sided valve surgery are as follows: In patients with severe TR who are undergoing left-sided valve surgery, tricuspid valve surgery is recommended as primary or functional severe TR does not consistently improve after treatment of a left-sided valve lesion. In patients with mild, moderate, or greater functional TR who are undergoing left-sided valve surgery, concomitant tricuspid valve repair is suggested if there is either (1) tricuspid annular dilation (diameter on transthoracic echocardiogram of > 40 mm or 21 mm/m^2^ indexed for body surface area; > 70 mm diameter on direct intraoperative measurement) or (2) prior evidence of right heart failure.

## Results

The mean age of the sample population of this study was 41 years (range 12-70) with a standard deviation of 12. There was a total of 73 male and 85 female patients. Twenty-two (22) patients (13.9%) underwent concurrent tricuspid annuloplasty with MVR, whereas 136 patients (86.1%) underwent no additional tricuspid interventions with MVR. Table 1 lists the general characteristics, comorbidities, and medications of patients in both groups.

**Table 1:** Patient characteristics, preoperative comorbidities, and medications MI- Myocardial Infarction; NYHA- New York Heart Association; sPAP- Systolic Pulmonary Artery Pressure; MR- Mitral Regurgitation; ACE- Angiotensin-Converting Enzyme; TA- Tricuspid Annuloplasty; MVR- Mitral Valve Replacement

	TA+MVR (n=22)	MVR only (n=136)
Age, yr, mean + SD	37 + 11	41 + 12
Female sex	14 (63.6%)	71 (52.2%)
Diabetes	3 (13.6%)	18 (13.2%)
Hypertension	10 (45.5%)	36 (26.5%)
Coronary artery disease	1 (4.5%)	8 (5.9%)
Previous MI	0	7 (5.1%)
Renal insufficiency	3 (13.6%)	5 (3.7%)
Chronic lung disease	0	4 (2.9%)
Atrial fibrillation	4 (18.2%)	8 (5.9%)
NYHA III/IV	12 (54.5%)	69 (50.7%)
sPAP, mmHg, mean + SD	50.5 + 22	47.5 + 26
Moderate-to-severe MR	17 (77.3%)	86 (63.2%)
Mitral stenosis	14 (63.6%)	70 (51.5%)
Medications		
Beta blockers	9 (40.9%)	56 (41.2%)
ACE inhibitors	3 (13.6%)	13 (9.6%)
Nitrates	6 (27.3%)	16 (11.8%)
Warfarin	3 (13.6%)	27 (19.9%)
Inotropes	0	1 (0.7%)
Aspirin	10 (45.5%)	45 (33.1%)
Statins	3 (13.6%)	15 (11%)

**Figure 1:**
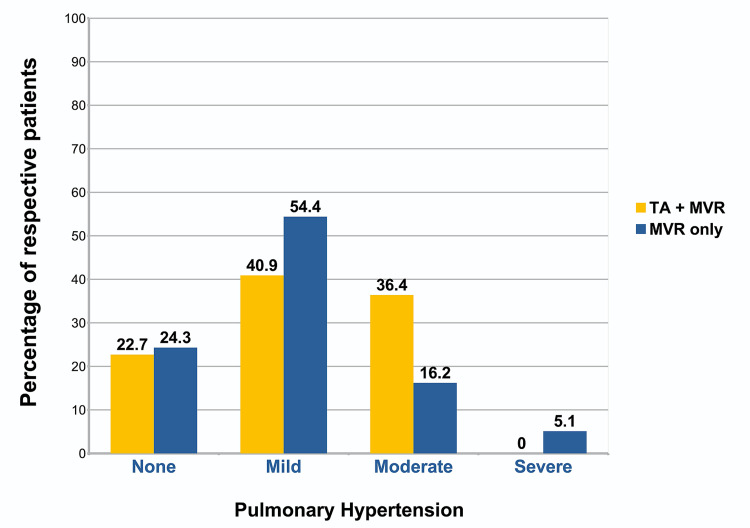
Pulmonary hypertension TA- Tricuspid Annuloplasty; MVR- Mitral Valve Replacement

Figure 1 illustrates the classification of patients in either group on the basis of pulmonary hypertension. Five patients in the TA+MVR group had no pulmonary hypertension compared with 33 patients in the MVR only group, nine vs 74 had mild, eight vs 22 had moderate, and zero vs seven had severe pulmonary hypertension, respectively. These are represented as the percentage of patients in either group and compared in Figure 1. The standard cut-offs for mean pulmonary artery pressure (mPAP) were used: mild = 25-40 mmHg; moderate = 41-55 mmHg; severe =>55 mmHg.

Ejection fraction in the given sample of patients was measured using preoperative echocardiography. Patients were subsequently classified in accordance with the ACC classification for ejection fraction, as shown in Table 2. The exceeding majority of patients in either group had a normal preoperative ejection fraction (LVEF=50%-70%).

**Table 2:** Ejection fraction TA- Tricuspid Annuloplasty; MVR- Mitral Valve Replacement

	TA+MVR (n=22)	MVR only (n=136)
Hyperdynamic (>70%)	0	0
Normal (50-70%)	19 (86.4%)	121 (89%)
Mild dysfunction (40-49%)	2 (9.1%)	13 (9.6%)
Moderate dysfunction (30-39%)	1 (4.5%)	2 (1.5%)
Severe dysfunction (<30%)	0	0

**Figure 2:**
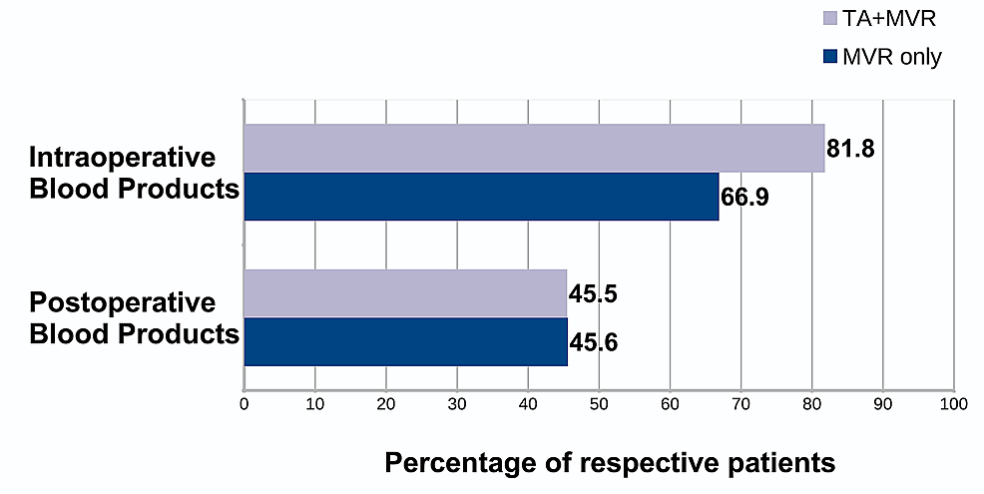
Blood products usage

Blood product usage was comparable between the two groups with no significant increase in either intraoperative (p=0.161) or postoperative (p=0.991) blood product requirement with the additional tricuspid procedure. Eighteen patients in the TA+MVR group required intraoperative blood products vs 91 patients in the MVR only group; 10 patients vs 62 patients required postoperative blood products, respectively. Blood products here refer to any of the following: red blood cells, fresh frozen plasma, cryoprecipitate, platelets, or whole blood. The proportions of patients in either group requiring blood products are shown in Figure 2.

The frequencies of in-hospital mortality as well as early postoperative complications in both groups are shown in Table 3. In-hospital mortality includes patients declared dead within the same hospitalization due to any cause, including those causes clearly unrelated to the operation. There was no significant increase in in-hospital death (4.5% vs 4.4%) associated with an additional TA procedure at the time of MVR. Likewise, simultaneous TA repair did not result in a significant increase in any of the early postoperative complications mentioned in Table 3. The mean length of hospital stay was similar between the two groups, and there was no incidence of postoperative renal failure prior to discharge in patients in either group. 

**Table 3:** Perioperative morbidity and mortality SD- Standard Deviation; TA- Tricuspid Annuloplasty; MVR- Mitral Valve Replacement

	TA+MVR (n=22)	MVR only (n=136)	p-value
In-hospital mortality	1 (4.5%)	6 (4.4%)	0.977
Prolonged ICU (>48 hrs)	3 (13.6%)	14 (10.3%)	0.639
Prolonged ventilation (>24 hrs)	0	3 (2.2%)	0.482
Re-intubation	2 (9.1%)	4 (2.9%)	0.161
Re-opening for bleeding tamponade	0	7 (5.1%)	0.276
Length of stay (days), mean + SD	6.34 + 3.5	5.1 + 3.1	0.100

## Discussion

Functional TR is a common finding in patients with mitral dysfunction such as those with rheumatic heart disease [12]. The pathophysiology is described as an increase in left atrial pressure translating into pulmonary hypertension. Long-term pulmonary hypertension increases afterload on the right ventricle, eventually leading to right ventricular dysfunction and remodeling. Ultimately, there is dilation of the tricuspid annulus, papillary muscle displacement, and tethering of leaflets of the tricuspid valve allowing regurgitant flow. Increased left atrial pressure may also lead to functional regurgitation by a secondary mechanism. Atrial fibrillation resulting from the enlarged left atrium can lead to dilation of the right atrium and, consequently of the tricuspid annulus, allowing regurgitation [2]. Less frequently, the tricuspid abnormality may be a product of organic tricuspid valve involvement by the rheumatic disease process itself. Indeed, most cases are likely a product of contribution from both mechanisms.

Despite the abundance of tricuspid dysfunction in mitral valve disease patients, tricuspid valve repair at the time of mitral surgery is relatively underutilized. This is, in part, due to the expectation that the improvement in mitral valve function may optimize hemodynamic parameters allowing for improved tricuspid function [4]. However, studies have shown that, in most cases, the correction of left-sided disease is insufficient to improve tricuspid dysfunction [13-14]. This is especially true for patients with moderate to severe tricuspid regurgitation. Indeed, tricuspid annular dilation secondary to increased left-sided pressure may actually be an irreversible phenomenon. Sadeghi et al. observed that in patients with chronic thromboembolic pulmonary hypertension that underwent pulmonary thromboendarterectomy, the dilated tricuspid annulus persisted despite resolution of pulmonary hypertension [15].

Late TR is also a common phenomenon following mitral valve surgery. Numerous studies have sought to identify independent risk factors associated with the development of late significant TR following left-sided valve surgery [7,16]. Zhu et al. conducted a meta-analysis of 3138 subjects identifying factors such as atrial fibrillation, enlarged atria, rheumatic etiology, long time from surgery onset, prior valve surgery, female gender, and others as important predictors of late TR development [17]. It is important, however, to always exclude a dysfunctional prosthetic mitral valve via echocardiography as a possible cause of late TR following left-sided surgery [18].

Considering the potential for TR progression and late TR development, tricuspid intervention should be an important consideration at the time of initial mitral valve surgery. Currently, there is little debate regarding the utility of this procedure in patients with moderate to severe TR or significant dilation of the tricuspid annulus. Studies have shown positive effects on right ventricular remodeling and outcomes following concurrent TA with mitral valve surgery [19-21]. In general, tricuspid repair is preferred over replacement. Singh et al. found repair to be preferable even in organic tricuspid valve involvement, reporting higher perioperative and mid-term risk with tricuspid replacement compared to tricuspid repair in a group of 250 patients [22]. However, in other patients with more severe organic disease tricuspid repair may not be a viable option and replacement should be performed.

To assess surgical risk with concurrent TA, we reviewed a number of patients (n=158) that underwent MVR for rheumatic heart disease over a period of three and a half years. Patients that satisfied criteria based on the degree of TR, as well as the diameter of the tricuspid annulus, underwent additional tricuspid annuloplasty (n=22). The main focus of this study was on the comparison of in-hospital surgical risk associated with this additional procedure alongside MVR and whether deferral of TA may be justified for this reason. Patients were matched for additional long-standing co-morbidities and medications with proportionate distribution among both groups. A majority of patients in both groups had mild pulmonary hypertension (mPAP = 25-40 mmHg) and a normal ejection fraction (LVEF= 50-70 %). After matching for comorbidities and preoperative risk, we found that TA in addition to MVR did not increase surgical risk in terms of early mortality or complications.

## Conclusions

In our retrospective analysis of rheumatic heart disease patients, concurrent TA with MVR does not appear to increase perioperative morbidity or mortality. TA at the time of MVR should be an important consideration in patients with additional tricuspid dysfunction. When indicated, simultaneous TA repair should not be deferred on the basis of potentially increased surgical risk.
